# Effects of hot-water extracts from *Ganoderma lucidum* residues and solid-state fermentation residues on prebiotic and immune-stimulatory activities in vitro and the powdered residues used as broiler feed additives in vivo

**DOI:** 10.1186/s40529-015-0097-3

**Published:** 2015-07-04

**Authors:** Yuh-Hwa Liu, Yin-Shiou Lin, Kuan-Ling Lin, Yeh-Lin Lu, Chao-Hsiang Chen, Mei-Yin Chien, Huey-Fang Shang, Shyr-Yi Lin, Wen-Chi Hou

**Affiliations:** 1 Division of Gastroenterology, Shin Kong Wu Ho-Su Memorial Hospital, Taipei, Taiwan; 2 Department of General Medicine, Taipei Medical University, Taipei, Taiwan; 3 Graduate Institute of Pharmacognosy, Taipei Medical University, Taipei, Taiwan; 4 Department of Pharmacy, Taipei Medical University, Taipei, Taiwan; 5 Ko Da Pharmaceutical Co. Ltd, Tao-Yuan, Taiwan; 6 Department of Microbiology and Immunology, Taipei Medical University, Taipei, Taiwan

**Keywords:** Arbor Acres broiler chicks, *Ganoderma lucidum* residues (GLR), Phagocytic, Natural killer cytotoxicity, Prebiotic activity, Probiotics

## Abstract

**Background:**

Large amounts of *Ganoderma lucidum* (GL) commercial products are provided in the worldwide market such as powders, tea bags, or capsules as dietary supplements which contained triterpenoids and/or polysaccharides. Therefore, it was estimated that several thousand tons of GL residues (GLR) are produced and discarded. For recycling uses, the aim of this study was to evaluate the benefits of two hot-water extracts from GLR (HWP_GLR) and solid-state fermentation GLR inoculated with GL mycelia (HWP_GLRF) on the growths of *Lactobacillus rhamnosus* and *Bifidobacterium longum*. The RAW264.7 cells were used to investigate the effects of HWP_GLR and HWP_GLRF on nitric oxide productions, phagocytic activities against FITC-labeled *E. coli*, and to lower lipopolysaccharide (LPS)-binding capacities. The powders of GLR and GLRF were used as additives in the commercial feeds for feeding broiler chicks in vivo to evaluate the immune-stimulatory and prebiotic activities.

**Results:**

HWP_GLR and HWP_GLRF with molecular size 5 to 8 kDa were showed to stimulate growths of *L. rhamnosus* and *B. longum*. It was found that in the presence of polymyxin B HWP_GLR and HWP_GLRF could stimulate nitric oxide productions, elevate phagocytic activities against FITC-labeled *E. coli*, and to lower lipopolysaccharide-binding capacities in RAW264.7 cells. The broiler chicks were selected for feedings in vivo. The 1-day-old chicks were fed commercial feeds for 1 week, and then were fed without or with 4 or 8 % of GLR and GLRF additives for 3 weeks. There was no significant weight difference among feeding groups. However, the phagocytosis and natural killer cytotoxicity in the peripheral bloods, and prebiotic activities of bifidobacteria in feces of GLR and/or GLRF groups were significantly different compared to the control (*P* < 0.05).

**Conclusions:**

The GLR, GLRF, and their hot-water extracts with beneficial activities could be processed as feed additives which could increase the waste-recycling.

## Background

*Ganoderma lucidum* (GL), known as “Ling-Zhi”, is one of valuable Traditional Chinese Medicines, which has long been used as a tonic and to prevent and treat various diseases in China and other Asian countries for over 2000 years (Lee et al. 2012a). GL possessed a broader range of biological activities, such as antitumor, hypoglycemic, immunomodulatory, antioxidant, anti-diabetic, anti-atherosclerotic, anti-inflammatory, and anti-HIV activities (Wachtel-Galor et al. 2004; Boh et al. 2007; Mahajna et al. 2008; Sanodiya et al. 2009). Further, the clinical investigations also revealed that GL polysaccharide extracts could significantly enhance immune responses in patients with advanced-stage cancer (Gao et al. 2003). Moreover, the bioactive components of GL have been identified as polysaccharides, proteins, alkaloids, steroids, nucleosides, and triterpenoids etc. (Kino et al. 1989; Shiao 1992; Lee et al. 2012a). In additions, several literatures have shown that the GL polysaccharide-rich fractions exhibited abilities to stimulate the productions of IL-1, IL-6, IL-12, IFN-γ, tumor necrosis factor-α, IL-6, colony stimulating factor, and nitric oxide, which resulted in the stimulation of splenocyte proliferation and macrophage phagocytosis (Ohno et al. 1998; Mao et al. 1999; Chen et al. 2004).

Recently, mushroom with abundances of chitins, hemicelluloses, and glucans (Manning and Gibson 2004) has attracted much attentions as potential sources of prebiotics (Aida et al. 2009). The prebiotics are termed as “nondigestible food ingredient that beneficially affects the host by selectively stimulating the growth and/or activity of one or a limited number of bacteria in the colon, and thus improves host health”, and intakes of prebiotics can significantly modulate the colonic microbiota by increasing the number of specific bacteria and thus changing the composition of the microbiota (Gibson and Roberfroid 1995). It is reported that lactobacilli, such as *Lactobacillus acidophilus*, *L. casei*, *L. delbruekii*, and bifidobacteria, such as *Bifidobacterium longum*, are commonly recognized as health-promoting bacteria in guts and can be stimulated growth by prebiotics (Gibson and Roberfroid 1995; Roberfroid et al. 2010). These health-promoting effects may include the modulation of the intestinal microflora, prevention of rotavirus-induced or antibiotic-associated diarrhea, beneficial effects on gastrointestinal tract inflammation, modulate the mucosal immune responses (Roberfroid et al. 2010; Gourbeyre et al. 2011). The water-soluble and alkali-soluble extracts from cultivated oyster mushroom which contained branched 1,3-β-D-glucans, 1,6-β-D-glucans, and linear 1,3-α-D-glucans showed the growth stimulation toward probiotics (Synytsya et al. 2009). It was reported that a large amount of GL commercial products are provided in the worldwide market such as powders, tea bags, or capsules as dietary supplements which contained triterpenoids and/or polysaccharides. Therefore, it was estimated that several thousand tons of GL residues (GLR) are produced and discarded. For recycling uses, the aim of this study was to evaluate the benefits of two hot-water extracts from GLR (HWP_GLR) and solid-state fermentation GLR inoculated with GL mycelia (HWP_GLRF) on the growths of *Lactobacillus rhamnosus* and *Bifidobacterium longum*. The RAW264.7 cells were used to investigate the effects of HWP_GLR and HWP_GLRF on nitric oxide productions, phagocytic activities against *E. coli*, and to lower lipopolysaccharide (LPS)-binding capacities. The GLR and GLRF were used as additives in the commercial feeds for feeding broiler chicks in vivo to evaluate the immune-stimulatory and prebiotic activities.

## Methods

### Materials

The *G. lucidum* BCRC 36041 and RAW 264.7 were obtained from the Bioresource Collection and Research Center (BCRC), Food Industry Research and Development Institute (Hsinchu, Taiwan). LSCC-RP9 cells (avian leukosis virus-transformed B-lymphoblastoid cell line from chickens) were kindly provided by Avian Disease and Oncology Laboratory, Agricultural Research Service, US Department of Agriculture (MI, USA). LPS, sulfanilamide, sodium nitrite, lipopolysaccharide-fluorescein isocyanate (FITC), polymyxin B-agarose resin, 3-(4,5-Dimethylthiazol-2-yl)-2,5-diphenyltetrazolium bromide (MTT), inulin, trypan blue, and propidium iodide (PI) were all purchased from Sigma Chemical Co. (St. Louis, MO, USA). Difco™ Lactobacilli MRS broth, Endo broth, and reinforced clostridial broth were from Becton Dickinson (USA). Polymyxin B, kanamycin, iodoacetic acid, nalidixic acid, and 2,3,5-triphenyltetrazolium chloride, and vancomycin were from Abbott Lab. (IL, USA). Agar Bacteriological was from Oxoid Ltd. (Basingstoke, England). The GLR and GLRF were provided by Ko Da Pharmaceutical Co. Ltd (Tao-Yuan, Taiwan). The GL raw materials were extracted by 12-fold boiling water (*w/v*) to produce commercial products, and the residues were then dried as GLR. The *G. lucidum* BCRC 36041 was maintained on potato dextrose agar slants, and the activated mycelia of *G. lucidum* BCRC 36041 were obtained in liquid medium following the previous method (Hsieh and Yang 2004). For GLRF production, the activated mycelia of *G. lucidum* BCRC 36041 in the potato dextrose broth were homogenized and inoculated onto the autoclaved GLR in the flask [about 10 % inoculum for GLR (*v/w*) in each container] and placed at 25 to 30 °C for solid-state fermentations.

### The HWP_GLR and HWP_GLRF preparations and β-glucan content determinations

Each GLR and GLRF (about 60 g) was weighted and placed in the bottle with screw cap and 600 ml distilled water was added, the mixture was autoclaved at 121 °C for 30 min. After filtration, each filtrate was lyophilized as HWP. The proximate compositions of GLR and GLRF were assayed by TÜV Rheinland AIMEX Ltd. (Pintung, Taiwan). The β-glucan contents of HWP_GLR and HWP_GLRF were determined according to the manufacturer’s instructions (K-BGLU 07/11, Megazyme International, Ireland) which were hydrolyzed by lichenase (a specific endo-(1–3)(1–4)-β-D-glucan 4-glucanohydrolase) and β-glucosidase and the released glucose was then determined by glucose oxidase/peroxidase reagents.

### HWP_GLR and HWP_GLRF molecular weight determinations

The molecular weights of HWP_GLR and HWP_GLRF were determined by using a Breeze HPLC system (Waters Co., Milford, MA) equipped with a 10 μl loop, a G4000PW_XL_ size exclusion column (7.8 × 300 mm, TSK-GEL, Tosoh Corp., Tokyo, Japan) at 55 °C, and a RI detector (oven, 50 °C). An aliquot of 10-μl (10 μg/μl) sample solution was injected and eluted with 0.1 M NaN_3_ aqueous solution (Karnjanapratum et al. 2012) at a flow rate of 0.5 ml/min. The calibration curve was prepared from dextran standards (1400, 670, 270, 50, 12 and 5 kDa).

### Effects of HWP_GLR and HWP_GLRF on the cell viability, nitric oxide production, phagocytosis, and LPS binding inhibitory activity in RAW264.7 cells

The RAW 264.7 macrophages were cultured in Dulbecco’s modified Eagle medium (DMEM), 10 % fetal bovine serum (FBS), and penicillin/streptomycin solution (10,000 units/ml of penicillin, 10 mg/ml of streptomycin). The cells were incubated at 37 °C in a humidified atmosphere with 5 % CO_2_. For cell viability assay, 4 × 10^5^/ml RAW 264.7 cells were seeded onto a 96-well microtiter plate, and various concentrations of HWP_GLR and HWP_GLRF (0.25, 0.5, 0.75 and 1 mg/ml) were added in the present of polymyxin B (50 μg/ml). Then, the cells were incubated at 37 °C in a humidified atmosphere with 5 % CO_2_ for 24 h. After the end of incubation, RAW 264.7 cells were incubated with 250 μg/ml of MTT for cell viability assay (Liu et al. 2007). For nitric oxide production assay, RAW 264.7 cells were seeded onto a 96-well microtiter plate and various concentrations of HWP_GLR and HWP_GLRF (0.25, 0.5, 0.75 and 1 mg/ml) in the presence of polymyxin B or LPS (600 ng/ml) were added with or without polymyxin B additions. The nitric oxide production was assayed by Griess reagents and the sodium nitrite (0–100 μΜ) was used to plot the calibration curve (Liu et al. 2008). For stimulated phagocytic activity of RAW264.7 cells toward FITC-labeled *E. coli*, the different concentration of HWP_GLR and HWP_GLRF (0.25, 0.5, 0.75 and 1 mg/ml) or LPS (600 ng/ml) were added to the cultured RAW264.7 cells in the presence or absence of polymyxin B following the previous method (Liu et al. 2007). For LPS binding inhibitory activity in RAW264.7 cells, each reagent used was passed through polymyxin B-agarose column and HWP_GLR and HWP_GLRF solutions were pre-mixed with polymyxin B-agarose resins in advance to remove LPS contaminations, then, HWP_GLR and HWP_GLRF (0.1 and 1 mg/ml) were added into RAW 264.7 cells for 15 min and then FITC-labeled LPS (200 ng/ml) were added for 1 h at 37 °C and analyzed by a BD FACSCanto II flow cytometry (BD Bioscience, USA) which the median fluorescence intensity was measured, and FITC-LPS binding to the RAW264.7 cells was recognized as 100 % (An et al. 2002).

### Bacterial enumeration after HWP_GLR and HWP_GLRF treatments in vitro

The 100 μl of each bacterial culture (grown in each selective broth) was pre-mixed with different concentrations of HWP_GLR and HWP_GLRF (1, 5, 10 mg/ml), diluted serially and then spread onto selective agar plate, and then incubated in an anaerobic chamber (Becton Dickinson, USA) at 37 °C for 48 h (*L. rhamnosus* GG and *B. longum*) or 24 h (*E. coli*). The selective growth medium for *L. rhamnosus* GG was MRS medium (Difco™ Lactobacilli MRS broth plus Agar Bacteriological. After being autoclaved, the filtered vancomycin was added and poured onto petri dishes for further uses); the selective growth medium for *B. longum* was BIM-25 medium (reinforced clostridial broth plus Agar Bacteriological. After being autoclaved, the filtered polymyxin B, kanamycin, iodoacetic acid, nalidixic acid and 2,3,5-triphenyltetrazolium chloride each was serially added and poured onto petri dishes for further uses); the selective growth medium for *E. coli* was Endo medium (Endo broth and Agar Bacteriological. After being autoclaved, the medium was poured onto petri dishes for further uses). The inulin (40 mg/ml) was used as a positive control. The bacterial colonies were counted and calculated, and expressed as growth stimulation index as following: (colony numbers of inulin or HWP_GLR or HWP_GLRF)/(colony numbers of the blank).

### Broiler chick feeding experiments in vivo

The feeding experiment was performed at Ko Da Pharmaceutical Co. Ltd (Tao-Yuan, Taiwan). All animal experimental procedures followed the published guidelines of National Science Council, Taipei, Taiwan (1994). The newborn Arbor Acres broiler chicks (*N* = 30, 1-day-old) were purchased from Arbor Acres Taiwan Co., Ltd. (Taipei, Taiwan) and had free access to newborn broiler chick feeds (Fwusow Industry Co. Ltd, Shalu, Taichung) and water for 1 week. These broiler chicks were randomly divided into five groups (*N* = 6, two chicks in one cage), the control group fed with medium-sized broiler chick feeds (Fwusow Industry Co. Ltd, Shalu, Taichung) and four groups fed with functional feeds, 4 or 8 % (*w/w*) of GLR and GLRF powders added in the medium-sized feeds, for 3 weeks. The broiler chicks were weighed every week. The feces of each group were collected at day 28 (the end of the feeding experiments). For lactobacilli and bifidobacteria enumeration determinations, the MRS and BIM-25 selective agar plates were used, respectively. The feces (about 0.05 g) were suspended in the autoclaved broth and diluted serially and spread onto selective agar plate, and then incubated in an anaerobic chamber at above-mentioned conditions. The bacterial colonies were counted and calculated, and expressed as log colony forming unit (CFU)/g feces. To evaluate the effects of feed additives on phagocytic activities of polymorphonuclear (PMN) cells and the natural killer (NK) activities of peripheral blood mononuclear cells (PBMC) in the peripheral blood, the blood samples were drawn from the chicken wings at the last week of the feeding experiments.

### Phagocytosis of polymorphonuclear cells in the peripheral blood

The FITC-labeled *E. coli* (Molecular Probes, USA) powder (5 mg) was suspended in 0.5 ml of Hank’s balanced salt solution (HBSS) and used for phagocytic analysis by flow cytometry (Liu et al. 2007; Shang et al. 2007). Eighty microliters of blood from the chicken wing were mixed with 20 μl of FITC-labeled *E. coli* solution at 37 °C for 10 min. The Falcon tube was immersed in an ice bath to stop the phagocytosis. Eighty microliters of trypan blue (1.25 mg/ml) were added to quench the residual FITC-labeled *E. coli*. After lysis and washing, 5 μl PI (2 mg/ml) were added for 10 min, and the phagocytosis of PMN cells was determined by flow cytometry (Becton Dickinson FACS Calibur™, CA).

### NK cytotoxicity of peripheral blood mononuclear cells in the peripheral blood

The LSCC-RP9 cells (avian leukosis virus-transformed B-lymphoblastoid cell line from chickens) were used as target cells of PBMC in the peripheral blood for NK cytotoxic activity assay by a flow cytometry using the DiOC/18 membrane dye (Molecular Probes, Eugene, OR) to stain alive LSCC-RP9 cells and PI nuclear dye to stain the dead cells (Kushima et al. 2003). The chicken PBMC were obtained by centrifuging the whole blood and a PBS mixture on Ficoll–Hypaque (2.4:1, V/V) by the density-gradient centrifugation. The cultural medium (2 l) for LSCC-RP9 cells included 11.9 g McCoy’s 5A medium modified (Sigma Co., USA), 13.8 g Leibowitz L-15 medium (Sigma Co., USA), 20 % chicken serum, 10 % fetal bovine serum, and 5 % tryptose phosphate broth. For NK cytotoxic activity assay, the LSCC-RP9 cells were adjusted to 2 × 10^6^ cell/ml by the cultural medium. Ten microliter of 3 mM DiOC_18_ were added into 1000 μl of target cells at 37 °C for 20 min and then were suspended in cultural medium for further uses. The PBMC (effector cells) were mixed with target cells at ratios of 20:1 and then were co-cultured in 5 % CO_2_ humidified incubator at 37 °C for 2 h. The supernatants were removed and the same volume of PI solution (0.2 mg/ml) was added. The NK cytotoxicity was determined by flow cytometry (Becton Dickinson FACS Calibur™, CA).

### Statistical analyses

All data were calculated as means ± SD and multiple group comparisons were performed using one-way ANOVA, followed by the post hoc Tukey’s test for comparisons. Values not sharing the same alphabetic letter were significantly different (*P* < 0.05). The statistical analysis was performed using the GraphPad Prism Software 5.0 (San Diego, CA, USA).

## Results

### The general properties of GLR, GLRF, and the hot-water extracts

**Fig. 1:**
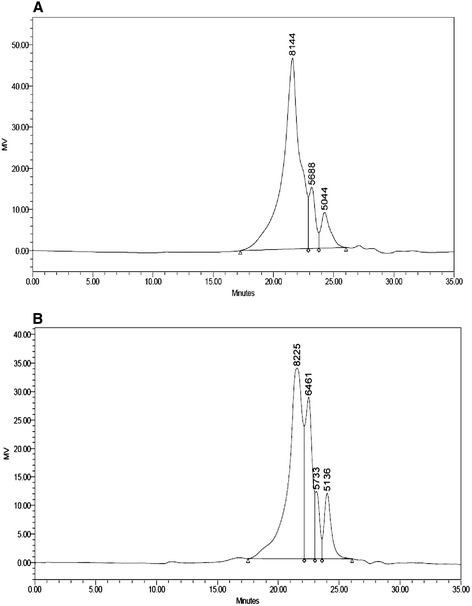
The size exclusion HPLC profiles of (**a**) HWP_GLR and (**b**) HWP_GLRF. The molecular sizes were determined by using a Breeze HPLC system (Waters Co., Milford, MA) equipped with a 10 μl loop, a G4000PW_XL_ size exclusion column (7.8 × 300 mm, TSK-GEL, Tosoh Corp., Tokyo, Japan) at 55 °C, and a RI detector (oven, 50 °C). An aliquot of 10-μl (10 μg/μl) sample solution was injected and eluted with 0.1 M NaN_3_ aqueous solution at a flow rate of 0.5 ml/min

The proximate compositions of GLR and GLRF were showed in Table 1. The GLR showed high amounts of crude fiber contents (41 %) and the nitrogen free extracts (NFE, 31.9 %). The crude fiber contents in GLRF were 22.4 %, and the NFE were calculated to be 46.1 %. The GLR and GLRF were suspended in distilled water and autoclaved at 121 °C for 30 min, filtered and lyophilized to get hot water extracts, HWP_GLR and HWP_GLRF, which the recovery was 4/100 g and 6.6/100 g, respectively. The Fig. 1 showed the size exclusion HPLC profiles of (a) HWP_GLR and (b) HWP_GLRF, and the percentage distributions of the molecular size in each HWP were showed in the Table 2. There were three peaks in HWP_GLR, 8144, 5688 and 5044 Da which accounted for 82.2, 9.6 and 8.3 % of total area, respectively; four peaks in HWP_GLRF, 8225, 6461, 5733 and 5136 Da which accounted for 58.9, 24.7, 6.4 and 10 % of total area. It seemed that parts of higher molecular size in HWP_GLR (such as 8.144 kDa) was shifted to medium size one (6461 Da) in HWP_GLRF after solid-state fermentations. Using enzymatic hydrolysis to determine the β-glucan contents in HWP, it was found that the HWP_GLRF was about 6-folds higher that of HWP_GLR.

**Table 1:** The proximate compositions of GLR and GLRF

Sample	Crude protein (g/100 g)	Crude fat (g/100 g)	Crude fiber (g/100 g)	Moisture (g/100 g)	Ash (g/100 g)	NFE^a^ (g/100 g)
GLR	10.9	0.6	41.0	13.0	2.6	31.9
GLRF	13.2	0.6	22.4	7.6	10.1	46.1

^a^ *NFE* (nitrogen free extracts) is calculated by 100 % − (crude protein + crude fat + crude fiber + ash + moisture)%

**Table 2:** The β-glucan contents and molecular weight distributions of HWP_GLR and HWP_GLRF

Parameter	HWP_GLR	HWP_GLRF
β-glucan contents (mg/g)	2.10	12.84
Molecular weight (distributions, area %)^a^	8144 Da (82.2 %)^a^	8225 Da (58.9 %)
	5688 Da (9.57 %)	6461 Da (24.7 %)
	5044 Da (8.27 %)	5733 Da (6.38 %)
		5136 Da (10.0 %)

^a^Calculated from Fig. 1

### Effects of HWP_GLR and HWP_GLRF on the cell viability, nitric oxide production, phagocytosis, and LPS binding inhibitory activity of RAW264.7 cells

**Fig. 2:**
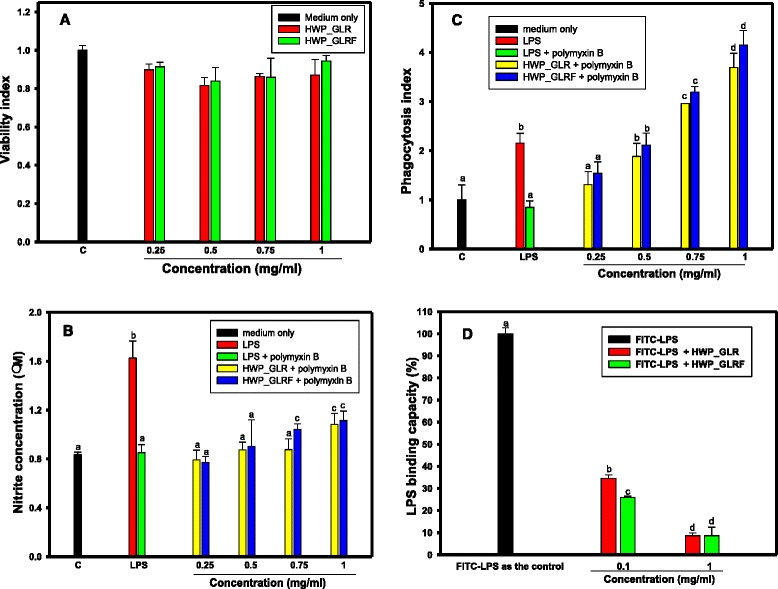
Effects of HWP_GLR and HWP_GLRF (0.25, 0.5, 0.75 and 1 mg/ml) on (**a**) the viability; (**b**) nitric oxide productions; (**c**) the phagocytosis; and (**d**) FITC-labeled LPS binding capacities of RAW 264.7 cells in the presence of polymyxin B (50 μg/ml). LPS with or without polymyxin B additions were used as the controls. Data were means ± SD and analyzed using one-way ANOVA, followed by the post hoc Tukey’s test for comparisons. Values not sharing the same *alphabetic letter* were significantly different (*P* < 0.05)

The HWP_GLR and HWP_GLRF showed no significant cytotoxicity among 0.25 to 1.0 mg/ml (Fig. 2a). The LPS showed significantly to stimulate the nitric oxide productions (*P* < 0.05) compared to the medium only (as the control), and this effect was abolished in the presence of polymyxin B (Fig. 2b) and showed no significant difference (*P* > 0.05) compared to the medium only (the control). Under non-toxic concentrations, HWP_GLRF at 0.75 and 1.0 mg/ml and HWP_GLR at 1 mg/ml in the presence of polymyxin B were showed to stimulate nitric oxide productions and significantly different to the LPS in the presence of polymyxin B (*P* < 0.05) (Fig. 2b). The HWP_GLR and HWP_GLRF (0.5, 0.75 and 1.0 mg/ml) exhibited dose-dependently to elevate phagocytic activities of the RAW264.7 cells against *E. coli* and showed significant differences compared to the control (medium only) (*P* < 0.05) (Fig. 2c). Using the FITC-labeled LPS as the positive controls, the pretreatments of HWP_GLR and HWP_GLRF at concentrations of 0.1 and 1.0 mg/ml could reduce LPS binding to RAW264.7cells and showed significant difference (*P* < 0.05) to the control (Fig. 2d).

### Prebiotic activity of HWP_GLR and HWP_GLRF in vitro

The effects of HWP_GLR and HWP_GLRF (5 and 10 mg/ml) on growths of *L. rhamnosus* GG, *B. longum*, and *E. coli* were showed in Table 3 and expressed as growth stimulation index (the blank was recognized as 1). Compared to the positive control of inulin (40 mg/ml), the HWP_GLR and HWP_GLRF showed better prebiotic activities and significant difference (*P* < 0.05) than those of inulin in the stimulatory growths of probiotics, *L. rhamnosus* GG and *B. longum*, but not the pathogenic *E. coli*, especial at the concentration of 10 mg/ml. Though HWP_GLRF showed higher β-glucan contents than HWP_GLR did (Table 2), the latter seemed to have higher capacity in stimulatory growths of *L. rhamnosus* GG and *B. longum* (Table 3).

**Table 3:** Effects of HWP_GLR, HWP_GLRF, and inulin on bacterial growths

Treatment	Growth stimulation index		
	*Lactobacillus rhamnosus GG*	*Bifidobacterium longum*	*Escherichia coli*
Blank	1.0 ± 0.4 a^#^	1 ± 0.07 a	1.0 ± 1.3 a
Inulin (40 mg/ml)	3.0 ± 0.2 b	3.3 ± 0.2 b	N.D.
HWP_GLR			
5 mg/ml	11.1 ± 4.5 c	0.8 ± 0.7 a	1.1 ± 1.1 a
10 mg/ml	83.2 ± 9.3 f	100.3 ± 22.0 d	3.4 ± 1.1 a
HWP_GLRF			
5 mg/ml	38.9 ± 7.0 d	5.1 ± 1.6 b	0.6 ± 0.6 a
10 mg/ml	67.2 ± 1.0 e	63.7 ± 9.9 c	2.2 ± 1.0 a

*N.D.* not detected

^#^All data were calculated as means ± SD and multiple group comparisons were performed using one-way ANOVA, followed by the post hoc Tukey’s test for comparisons. Values not sharing the same alphabetic letter were significantly different (*P* < 0.05)

### Broiler chick feeding experiments in vivo

**Fig. 3:**
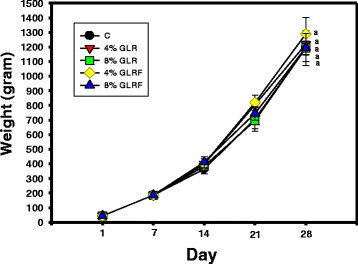
Effects of GLR or GLRF additives (4 or 8 %) in the commercial feeds on weights of Arbor Acres broiler chickens. The newborn Arbor Acres broiler chicks (*N* = 30, 1-day-old) were purchased from Arbor Acres Taiwan Co., Ltd. and had free access to newborn broiler chick feeds and water for 1 week. These broiler chickens were randomly divided into five groups, the control group fed with medium-sized broiler chick feeds and four groups fed with functional feeds, 4 or 8 % (*w/w*) of GLR and GLRF powders added in the medium-sized feeds, for 3 weeks

**Fig. 4:**
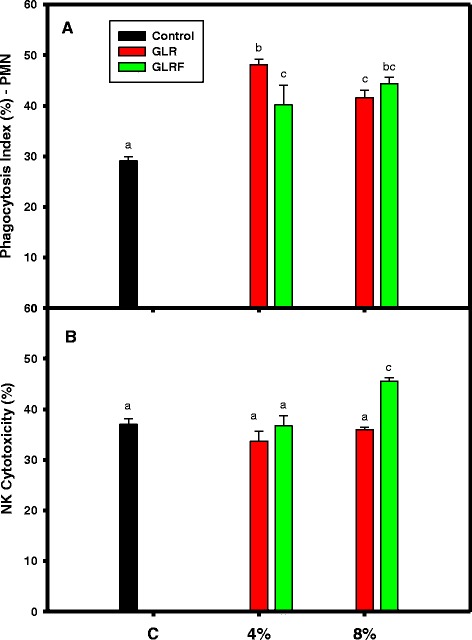
Effects of powders of GLR or GLRF additives (4 or 8 %) on the (**a**) phagocytic activities of polymorphonuclear (PMN) cells and (**b**) natural killer (NK) activities of peripheral blood mononuclear cells in the peripheral blood. The blood samples were drawn from the Arbor Acres broiler chicken wings at the last week of the feeding experiments. Data were means ± SD and analyzed using one-way ANOVA, followed by the post hoc Tukey’s test for comparisons. Values not sharing the same *alphabetic letter* were significantly different (*P* < 0.05)

Therefore, the 4 or 8 % of powdered GLR and GLRF added in the commercial medium-sized broiler chick feeds were used for 3-week feeding experiments. During feeding periods, the weights of Arbor Acres broiler chickens among groups were not shown significantly different (Fig. 3), however, the phagocytic activity of PMN in GLR or GLRF groups (4 or 8 % additives) was elevated and showed significantly different compared to the control (*P* < 0.05) (Fig. 4a). For the 4 % additives, the PMN in GLR group showed higher phagocytic activity than that of GLRF group (*P* < 0.05). For the 8 % additives, the PMN in both groups showed similar phagocytic activities. It was found that only 8 % GLRF additives could elevate NK activities of PBMC and showed significantly different compared to the control (*P* < 0.05) (Fig. 4b). The present results showed that GLR or GLRF additives in the feeds could stimulate the innate immunity of Arbor Acres broiler chickens against microbial invasions.

**Fig. 5:**
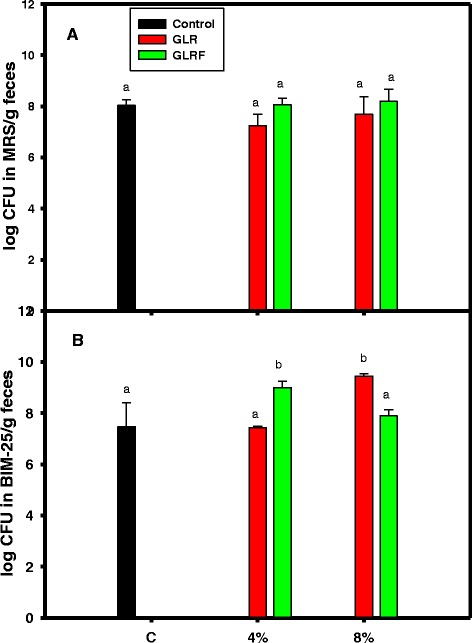
Effects of powders of GLR or GLRF additives (4 or 8 %) on the growths of probiotics in vivo. (**a**) Lactobacilli counts by uses of MRS selective agar plates and (**b**) bifidobacteria counts by uses of BIM-25 selective agar plates. The feces (about 0.05 g) were suspended in the autoclaved broth and diluted serially and spread onto selective agar plate, and then incubated in an anaerobic chamber. The bacterial colonies were counted and calculated, and expressed as log colony forming unit (CFU)/g feces. Data were means ± SD and analyzed using one-way ANOVA, followed by the post hoc Tukey’s test for comparisons. Values not sharing the same *alphabetic letter* were significantly different (*P* < 0.05)

The effects of GLR or GLRF additives on the growths of probiotics were showed in the Fig. 5. The MRS selective agar plate was used to count the lactobacilli (Fig. 5a) and the BIM-25 selective agar plate was used to count the bifidobacteria (Fig. 5b). It was found that the bifidobacteria, but not the lactobacilli, showed higher counts and significant differences compared to the control (*P* < 0.05) of the 4 % GLRF or 8 % GLR additives in the feeds.

## Discussion

The large amounts of triterpenoid-contained or polysaccharide-contained or enriched GL commercial products with an annual global value over $1.5 billion were available in the markets (Liu et al. 2010). It was estimated that several thousand tons of GLR were discarded. It was previously reported that the processed product (sacchachitin) from residues of *G. tsugae*, a copolymer of 60 % β-1,3-glucan and 40 % poly(*N*-acetylglucosamine), was applied as skin substitutes (Su et al. 1997). In the present study, the wastes of GLR or the solid-state fermented GLR (GLRF) as broiler chick feed additives (such as 4 % additives) exhibited prebiotic (Fig. 4) and innate immune-stimulatory activities (Fig. 5) in vivo which might increase the recycling uses in the future. Chang and Lu (2004) reported that HWP from GL fruiting bodies (designed as GLG, GLPS-2, and GLPS-3 in eluted orders with 0.3 M NaN_3_ by size exclusion HPLC equipped with the RI detector) contained mainly glucose and galactose and less amounts of mannose constituents. The GLG contained the aniline blue-positive β-glucans with average molecular mass of 2080 kDa; the GLPS-2 and GLPS-3 contained less amount of aniline blue-positive β-glucans with average molecular mass of 230 kDa and 120 kDa, respectively. These three fractions (GLG, GLPS-2, and GLPS-3) showed immune-stimulatory activities toward TNF-α secretions in human blood mononuclear cells in orders of GLG > > GLPS-2 > > GLPS-3. Liu et al. (2010) reported two low-molecular-weight glucans (GLP_L_1 and GLP_L_2) isolated from HWP of GL fruiting bodies by ion exchange chromatography and gel filtration with average molecular weight of 5.2 and 15.4 kDa, respectively. The GLP_L_1 contained glucose with four major and one minor linkages in the percentage ratio of 21.9:20.3:23.7:24.0:3.7 of (a) → 3)-Glcp-(1→, (b) → 4)-Glcp-(1→, (c) → 6)-Glcp-(1→, (d) → 3,6)-Glcp-(1→, and (e) → 4,6)-Glcp-(1→. In the present results, using the size exclusion HPLC equipped with the RI detector and the same NaN_3_ eluent, a smaller molecular size of 5 to 8 kDa HWP were found in GLR and GLRF (Fig. 1) which were apparently different from that of Chang and Lu (2004) reported, but closed to GLP_L_1 of Liu et al. (2010) reported from fruiting bodies of *G. lucidum*. The inulin (β-2,1-fructan) was recognized as prebiotic substances (Aida et al. 2009) and used in the present research as the positive control (Table 3). The HWP_GLR and HWP_GLRF (10 mg/ml) showed much better than inulin (40 mg/ml) in stimulatory growths on *L. rhamnosus* GG and *B. longum* (Table 3). The β-glucan content in the present assay was based on lichenase and β-glucosidase hydrolysis for mixed-linkage of [(1–3)(1–4)]-β-D-glucan determinations. The amounts of detected β-glucan in HWP_GLRF were higher than HWP_GLR (Table 2), however, both of the stimulatory growths toward *L. rhamnosus* GG and *B. longum* (Table 3), and nitric oxide productions (Fig. 2b), phagocytic activities (Fig. 2c), and reduced LPS binding capacities (Fig. 2d) in RAW264.7 cells in the presence of polymyxin B in vitro were similar. It meant that the HWP_GLRF showed to increase the hot-water soluble β-glucan contents (Table 2), however, these increased glucans might be biological inactive in RAW264.7 cells and needed further investigations. Macrophages released several mediators, including inflammatory cytokines, IL-1, IL-6 and TNF-α, and nitric oxide which could induce the activation and differentiation of lymphocytes and the proliferation of granulocytes (Nathan 1987). In mammals, phagocytosis was a very important defense against pathogen invasions and apoptotic cell scavenging which were performed by phagocytes like macrophages, dendritic cells, and granulocytes (Stuart and Ezekowitz 2005). The LPS might bind to the Toll-like receptor 4 in the macrophages to activate downstream signaling pathway, such as NF-κB-mediated cytokine expression including IL-1, IL-6, TNF-α, and IFN-γ inflammatory cytokines (Akira 2003), and the polymyxin B could interact with LPS to retard binding to Toll-like receptor 4 and further inflammation. Therefore, the reduced LPS-binding capacities (Fig. 2d), elevated nitric oxide productions (Fig. 2b) and phagocytic activities (Fig. 2c) of HWP_GLR and HWP_GLRF were beneficial to innate immune responses.

Some reports were investigated on the improvement of feed properties in order to elevate the growth and/or meat qualities of chicken, such as the mushroom stalk residue additives in feeds could improve the oxidative status and meat quality of broiler chickens (Lee et al. 2012b), and the *Echinacea purpurea* L. additives in feeds could improve the oxidative status and meat quality in Arbor Acres broilers (Lee et al. 2013). The weights of broiler chicken among feeding groups did not show significant difference (Fig. 3), however, the phagocytosis and NK cytotoxicity (Fig. 4) of the innate immune in the peripheral blood and the counted probiotics of bifidobacteria fed with GLR and/or GLRF additives were apparently elevated compared to the commercial feeds (Fig. 5). It meant that the 4 or 8 % GLR and/or GLRF additives in the commercial feeds did not apparently influence the outcomes of chicken weights during developments, but might enhance some physiological properties, such as prebiotic activities and immune responses.

## Conclusion

In conclusion, from the present in vitro and in vivo experiments, the GLR, GLRF, and their hot-water extracts with prebiotic and immune-stimulatory activities could be processed as feed additives which could increase the waste-recycling. It might be possible to add anti-bacterial properties of herbal medicines with GLR or GLRF to develop the new functional feeds with prebiotic, immune-stimulatory and anti-bacterial activities to reduce the uses of antibiotics and needed further investigations.
